# Complications arising from transfemoral, percutaneous implantation of an indwelling port–catheter system for hepatic infusion chemotherapy: Case series of the management and salvage of the system

**DOI:** 10.1016/j.ijscr.2019.10.017

**Published:** 2019-10-18

**Authors:** Misato Ueda, Kenshin Sai, Takashi Sonoda, Mina Tanaka, Yoshie Shibaoka

**Affiliations:** a Department of Plastic and Reconstructive Surgery, Meiwa Hospital, Japan; b Department of Medical Oncology, Meiwa Hospital, Japan; c Department of Plastic and Reconstructive Surgery, Rokko Island Konan Hospital, Japan

**Keywords:** Hepatic arterial infusion of chemotherapy, Implantable port–catheter system, Salvage, Case report

## Abstract

**Introduction:**

Regional hepatic arterial infusion of chemotherapy is performed for unresectable liver tumors via percutaneously implanted port–catheter systems; while these port–catheter systems are effective administration routes, they are associated with various complications. Withdrawal of the system is considered if the complications occur, but repeated hepatic arterial infusion of chemotherapy (HAIC) via an implanted port–catheter system is a last-resort treatment for unresectable advanced liver cancer, and the treatment must be continued. We discuss various cases with complications arising in the indwelling port area in hepatic arterial infusion of chemotherapy and report whether the system was salvaged.

**Methods:**

Between August 2013 and October 2017, eight patients (six males and two females) aged 61–80 years (mean age 76.6 years) with complications arising in a transfemoral indwelling port site for HAIC were referred to our department. All patients requested preservation of the system, especially the catheter. Each patient was assessed for the presence of “gross infection” based on a comprehensive evaluation of clinical findings and blood test results. In cases of “no gross infection,” we performed catheter salvage procedures.

If there was no clinical improvement following the catheter salvage procedure, the port–catheter system was withdrawn. This research work has been reported in line with the PROCESS criteria.

**Results:**

The port–catheter systems were withdrawn in two patients: one due to lasting infection and the other due to ulcer recurrence. Three cases were treated by removal of hematoma through an incision and ointment. The system was withdrawn in one of these cases due to exacerbation of ulcer; thus, the catheters were salvaged in five patients. None of these five patients experienced a relapse from 3 months to over 1 year after the procedure.

**Conclusion:**

The success of subcutaneous HAIC significantly impacts a patient’s prognosis, especially for unresectable tumors and residual tumor recurrences. Initially, we chose to preserve the devices without removal, particularly if there was no infection. However, this approach led to a delay in chemotherapy, prolongation of healing time, and additional complications. These cases demonstrate the importance of a thorough consultation with the patient’s oncologist to discuss whether or not the device should be conserved.

## Highlights

• We assessed complications arising from the transfemoral, percutaneous implantation of an indwelling port–catheter system and described salvage of the system.
• The catheter was salvaged by translating the subcutaneous pocket and changing the port and connecter or by removing the hematoma through an incision and applying ointment.
• Salvage of the system may cause additional complications. Therefore, follow-up examination and a thorough consultation with an oncologist are required.

## 1 Introduction

**Fig. 1:**
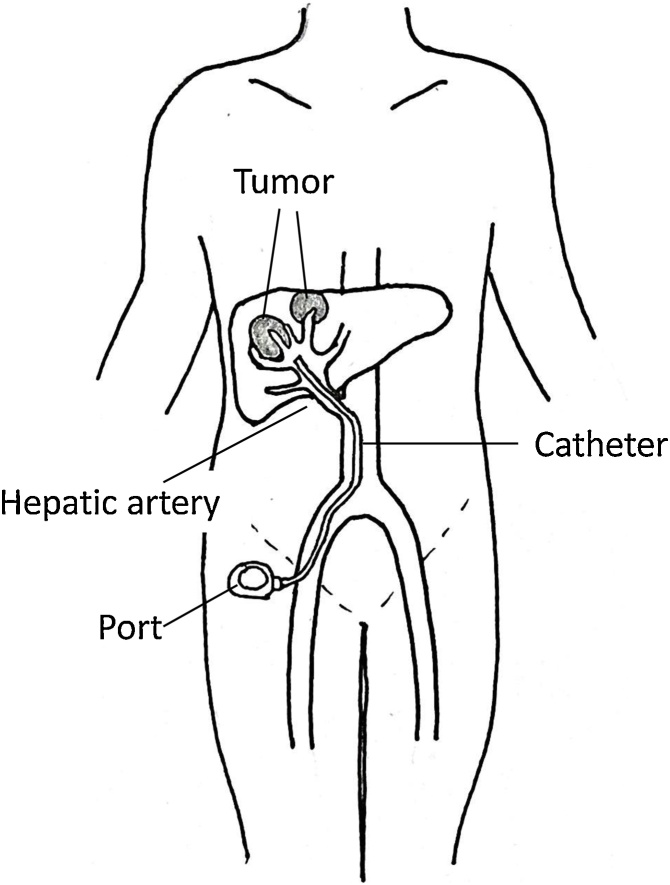
Hepatic arterial infusion chemotherapy with an implantable, transfemoral port–catheter system.

**Fig. 2:**
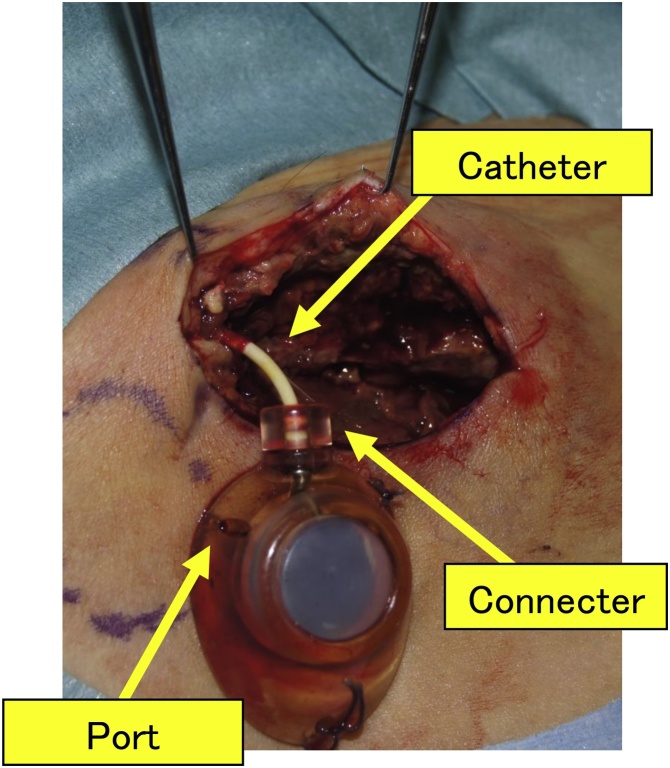
A port–catheter system comprises a catheter, connecter, and port.

**Fig. 3:**
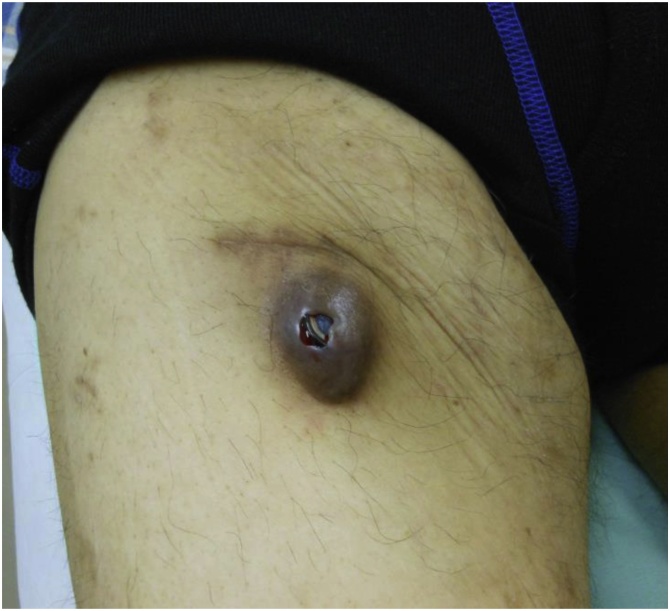
Exposure of the port due to skin defects.

Regional hepatic arterial infusion of chemotherapy (HAIC) optimizes the first-pass effects of cytotoxic agents, delivering high local drug concentrations to unresectable liver tumors with few significant systemic side effects. HAIC produces better response rates than systemic chemotherapy and is an important treatment option in patients with advanced, inoperable primary or metastatic hepatic tumors [1]. In HAIC, the transfemoral implantation of port–catheter systems accessed via the common femoral artery through the groin can effectively deliver drugs to the tumor (Fig. 1). A port–catheter system comprises three parts: the catheter, connecter, and port (Fig. 2). Drugs may be administered percutaneously through the puncture site of the port (Fig. 3).

HAIC is highly effective for cancer treatment, but it often exhibits various complications associated with the port–catheter system. These complications include system exposure or dislocation, hematoma, port infection, catheter infection, perforation, and dissection [2]. Major complications associated with the catheter or hepatic artery require withdrawal of the system. However, repeated HAIC via an implanted port–catheter system is a last-resort treatment for unresectable advanced liver cancer [3], and the treatment must continue. Because catheter replacement is typically difficult, salvage of the catheter is preferred. The system, especially the catheter, is often salvageable in cases of minor complications localized to the indwelling port area, such as exposure of the port due to skin defects, hematomas at the puncture site, and minor port infections. Here we discuss various cases with complications arising in the indwelling port area in HAIC and report whether the system was salvaged.

## 2 Methods

Between August 2013 and October 2017, eight patients (six males and two females) aged 61–80 years (mean age 76.6 years) with complications arising in a transfemoral indwelling port site for HAIC were referred to our department. All patients requested preservation of the system, especially the catheter. The underlying disease was hepatocellular carcinoma in three cases and hepatic metastases in five cases. The reasons for consultation were as follows: hematoma at the puncture site (n = 6), exposure of the port (n = 2), skin necrosis and ulcer (n = 3), and/or local infection (n = 1). Each patient was assessed for the presence of “gross infection” based on a comprehensive evaluation of clinical findings and blood test results. In cases of “no gross infection,” we performed catheter salvage procedures.

The catheter salvage procedure involved complete debridement and irrigation, changing of the port and connecter, preparing a new subcutaneous pocket in a healthy area, and implantation of the port in the new area. We ensured that the subcutaneous pocket contained adequate subcutaneous tissue. Skin defects were covered with simple sutures or local random flaps. If there was no clinical improvement following the catheter salvage procedure, the port–catheter system was withdrawn.

## 3 Results

The port–catheter systems were withdrawn in two patients: one due to lasting infection and the other due to ulcer recurrence. Three cases were treated by removal of hematoma through an incision and ointment. The system was withdrawn in one of these cases due to exacerbation of ulcer; thus, the catheters were salvaged in five patients (Table 1). None of these five patients experienced a relapse from 3 months to over 1 year after the procedure.

**Table 1:** Patient characteristics, treatment, and results.

Patient	Age (years) Sex	Primary disease	Complication	Treatment	Result
1	68 M	Hepatocellular cancer	Skin necrosis, port exposure	Change the connecter and port Pocket translation	Salvage of catheter
2	80 F	Hepatocellular cancer	Port exposure	Change the connecter and port Pocket translation	Salvage of catheter
3	78 M	Pancreatic cancer	Ulcer, hematoma	Change the connecter and port Pocket translation	Port infection Sepsis Removal of system
4	61 M	Stomach cancer	hematoma	Change the connecter and port Pocket translation	Salvage of catheter
5	75 M	Hepatocellular cancer	Skin necrosis, hematoma	Change the connecter and port Pocket translation	Catheter exposure Removal of system
6	67 F	Pancreatic cancer	Hematoma	Removal of hematoma Apply ointment	Salvage of catheter
7	69 M	Pancreatic cancer	Hematoma	Removal of hematoma Apply ointment	Salvage of catheter
8	68 M	Colorectal cancer	Hematoma local minor infection	Removal of hematoma Apply ointment	Salvage of catheter

## 4 Representative case report and surgical procedure

**Fig. 4:**
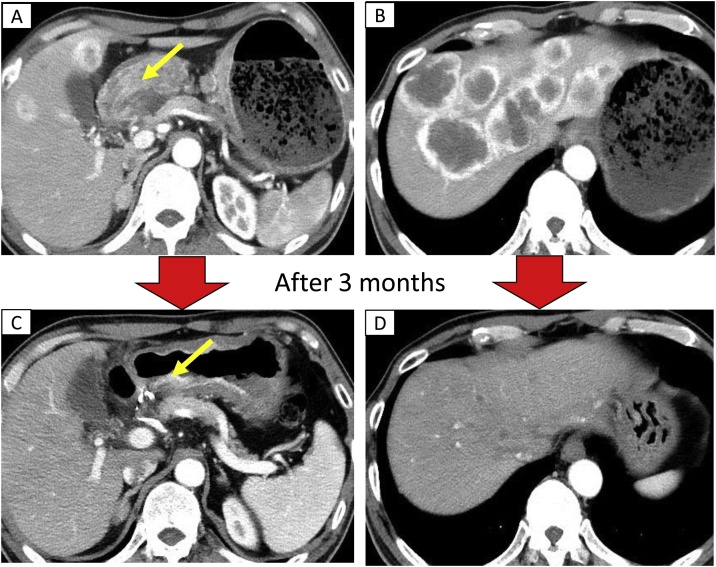
Efficiency of hepatic arterial infusion of chemotherapy (HAIC). (A) Pyloric obstruction due to stomach cancer (pointed by the arrows). (B) Liver metastasis. (C) 3 months after HAIC began, stomach tumor decreased in size and pyloric obstruction disappeared. (D) 3 months after the beginning of HAIC, the liver metastases greatly decreased.

**Fig. 5:**
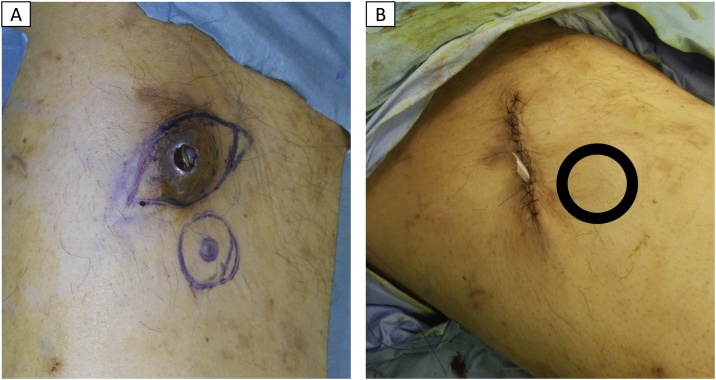
(A) Debridement of ulcer and capsule. (B) Port was replaced in the new pocket (circle) after exchanging the connecter and port.

A 61-year-old man with unresectable liver metastases from gastoric cancer presented with exposure of the port due to a skin defect. Hematoma had formed at the puncture site 8 months after the implantation of the port–catheter system, following which the port had become exposed. The HAIC treatment was highly effective, and the patient’s computed tomography scan showed a reduction in the size and metastases of the gastric cancer (Fig. 4). Salvage of the port–catheter system was requested due to the necessity for HAIC continuation, and he was referred to our department by a medical oncologist. We performed debridement of the ulcer, followed by complete capsulectomy and refreshment of the wound, and ensured adequate irrigation. The catheter was salvaged, and the connecter and port were changed. We prepared a new subcutaneous pocket with adequate subcutaneous fat tissue for the port in a healthy region. The port was implanted into the new pocket and the wound sutured (Fig. 5). Thus, the catheter was fully salvaged, and repeated HAIC could be continued; he survived for another 27 months.

## 5 Discussion

HAIC is a treatment that significantly impacts a patient’s prognosis. HAIC is performed in uncontrollable liver tumors, such as advanced cancer, vascular invasion, and multiple liver metastases. HAIC delivers high local drug concentration to unresectable liver tumors with few significant systemic side effects; however, various complications are associated with the port–catheter system [1]. The reported rate of complications associated with these systems is 7.8%–37% [[1], [2], [3], [4]]. When complications arise, removal of the port–catheter system may be necessary; however, the removal interrupts and prolongs chemotherapy, which may worsen a patient’s medical condition. HAIC via an implanted port–catheter system is a last-resort treatment for some patients with unresectable liver cancer, and the treatment must continue in them [[5], [6], [7]]. Moreover, replacement of the catheter is difficult in certain cases. Thus, salvage of the system, especially the catheter, is strongly suggested.

In cases such as bacteremia due to catheter infection, pseudoaneurysm or embolism due to catheters, and broken devices, removal of the whole system is required. The system, especially the catheter, is often salvageable in cases of minor complications localized to the indwelling port area, such as exposure of the port due to skin defects, hematomas at the puncture site, and minor port infections. We salvaged the catheter after ensuring that there was no gross infection, which was defined by a comprehensive evaluation of clinical findings and blood test results. If there was no improvement in complications after the local treatment, changing of the connecter and port, and translation of the subcutaneous pocket to a healthier area, the catheter was withdrawn from the patient.

However, catheter salvage may cause additional complications; thus, the possibility of salvage should be assessed for each patient individually. Several studies have reported on the salvage of exposed subcutaneous implantable devices, including pacemakers, venous access ports, and neural stimulators [[8], [9], [10], [11], [12]]. Regarding pacemakers, some authors advocate removal and delayed replacement when a clinical infection is evident [11,12]. However, some authors suggest immediate replacement of exposed devices in the absence of a gross infection [8,9]. Toia et al. [13] have described salvage of subcutaneous implantable devices (venous access port, cardiac pacemakers, and subcutaneous neural stimulators) with submuscular placement and immediate replacement. Removal and delayed replacement may cause chemotherapy delay, impaired prognosis, healing prolongation, and acute cardiac problems. Accordingly, our cases could have exhibited delayed chemotherapy and advancement of the cancer. Thus, we attempted salvaging the port–catheter system, especially the catheter.

When assessing the possibility of salvaging a catheter, it is important to first confirm the absence of an active infection; it is also important to consider the timing of the treatment. In cases where exposure of the system continues, induction of more gross infection, such as catheter infection and sepsis is expected.

Reportedly, catheter infection tends to occur later than port infection [2]. For salvaging the catheter, local infections and complications that potentially cause infection in the port portion must be ameliorated. In catheter salvage involving the exchange of ports and connecters, adequate debridement is necessary. Toia et al. [13] have reported that complete capsulectomy and adequate debridement of the pocket are mandatory for salvaging subcutaneous implantable devices. Following this, a new pocket is prepared in healthy tissue and coverage to the port is provided with adequate blood flow and thick subcutaneous tissue. Depending on the defect size and tightness of the skin, a local flap is also used for coverage.

Despite these carefully planned procedures, recurrence of complications may occur and pathologies may get exacerbated due to the salvage of port–catheter systems. Thus, proper judgment of patients’ conditions and the timing of treatment are of considerable importance. Furthermore, adequate follow-up is necessary, as salvage of the system may cause sepsis. A thorough consultation with a designated oncologist on a surgical team is required.

## 6 Conclusion

The success of subcutaneous HAIC significantly impacts a patient’s prognosis, especially for unresectable tumors and residual tumor recurrences. Initially, we chose to preserve the devices without removal, particularly if there was no infection. However, this approach led to a delay in chemotherapy, prolongation of healing time, and additional complications. These cases demonstrate the importance of a thorough consultation with the patient’s oncologist to discuss whether or not the device should be conserved.

## Funding

No sponsorship for this case reports.

## Ethical approval

This is case reports. Therefore, it did not require ethical approval from ethics committee. However, we have got permission from the patient.

## Consent

Written informed consents were obtained from the patients.

## Author’s contribution

Misato Ueda: participation in the treatment, data collection, and writing this manuscript. Kenshin Sai: participation in the treatment, follow-up the patients. Takashi Sonoda: participation in the treatment, follow-up the patients. Mina Tanaka; participation in the treatment. Yoshie Shibaoka: participation in the treatment, follow-up the patients.

## Registration of research studies

Research Registry Unique Identifying Number is researchregistry5036.

Public and Scientific Title of Research is “Complications arising from transfemoral, percutaneous implantation of an indwelling port–catheter system for hepatic infusion chemotherapy: Case series of the management and salvage of the syste”.

## Guarantor

Misato Ueda, M.D. Kenshin Sai, M.D., Ph.D.

## Provenance and peer review

Commissioned, externally peer-reviewed.

## Declaration of Competing Interest

The authors declare no conflicts of interest associated with this manuscript.
